# AI-Enabled Dynamic Edge-Cloud Resource Allocation for Smart Cities and Smart Buildings

**DOI:** 10.3390/s25247438

**Published:** 2025-12-06

**Authors:** Marian-Cosmin Dumitru, Simona-Iuliana Caramihai, Alexandru Dumitrascu, Radu-Nicolae Pietraru, Mihnea-Alexandru Moisescu

**Affiliations:** Faculty of Automatic Control and Computers, National University of Science and Technology Politehnica Bucharest, RO-060042 Bucharest, Romania; simona.caramihai@upb.ro (S.-I.C.); alexandru.dumitrascu@upb.ro (A.D.); radu.pietraru@upb.ro (R.-N.P.); mihnea.moisescu@upb.ro (M.-A.M.)

**Keywords:** resource allocation, edge computing, smart city, smart building

## Abstract

The rapid expansion of IoT devices represents significant progress in areas such as smart buildings and smart cities, but at the same time, the volume of data generated represents a challenge, which can lead to real bottlenecks in the data analysis process, thus resulting in increased waiting times for end users. The use of cloud-based solutions may prove inefficient in some cases, as the bandwidth required for transmitting data generated by IoT devices is limited. The integration with Edge computing mitigates this issue, bringing data processing closer to the resource that generates it. Edge computing plays a key role in improving cloud performance by offloading tasks closer to the data source, optimizing resource allocation. Achieving the desired performance requires a dynamic approach to resource management, where task execution can be prioritized based on current load conditions: either at the Edge node or the Cloud node. This paper proposes an approach based on the Seasonal Auto Regressive Integrated Moving Average (SARIMA) model for seamlessly switching between the Cloud and Edge nodes in the event of a loss of connection between the Cloud and Edge nodes. Thereby ensuring the command loop remains closed by transferring the task to the Edge node until the Cloud node becomes available. In this way, the prediction that could underlie a command is not jeopardized by the lack of connection to the cloud node. The method was evaluated using real-world resource utilization data and compared against a Simple Moving Average (SMA) baseline using standard metrics: RMSE, MAE, MAPE, and MSE. Experimental results demonstrate that SRIMA significantly improves prediction accuracy, achieving up to 64% improvement for CPU usage and 35% for RAM usage compared to SMA. These findings highlight the effectiveness of incorporating seasonality and autoregressive components in predictive models for edge computing, contributing to more efficient resource allocation and enhanced performance in smart city environments.

## 1. Introduction

The smart city concept is based on a city redefined by information and communication technology [1]. Based on this definition, it can be stated that the purpose of the smart city is to help its inhabitants in their daily activities through technology. At the same time, the smart building concept integrates the elements of intelligence, control, materials and construction into a cohesive building system. It is not limited only to the installation of sensors that transmit data, but also to making decisions based on the user’s behavior and their preferences for temperature, humidity, and the way to use certain functions [2].

A very important role in the development of these concepts is played by IoT devices and data-generating devices, devices that represent an innovative communication paradigm [3], which, according to [4], currently amount to approximately 19.8 billion; this number is expected to reach 40.6 billion in 2034, according to the forecast. Currently, the volume of data generated by IoT devices is approximately 90.3 Zettabytes (ZB) [5], a huge volume of data that must be processed, stored, and analyzed so that relevant decisions can be made in an optimal time. This growth is also reflected in the interest that has emerged for IoT integration within smart cities, with IEEE publishing over four hundred articles annually in this field [6].

In order to facilitate the processing and processing of data as quickly as possible, without having to transmit the data to the Cloud provider, Edge computing is introduced as the paradigm that brings resources responsible for computing and storage closer, at the edge of the network, closer to the IoT devices [7]. Thus, Edge computing is the main layer where data is sent, serving as an intermediate layer between the sensors that generate the data and the cloud, where all the data generated by the sensors is transmitted.

Unlike certain uses such as the healthcare field [8] where continuous monitoring of patient information is needed, with tasks being monitored and prioritized based on clear parameters in the patient’s file, within smart cities the research carried out addresses various topics that can be improved, most of which are focused on improving the security of data and city residents [9], managing the energy consumption required for systems [10], water and waste management [11] or traffic prediction [12]. Starting from a smaller scale within a smart building or integrating multiple buildings to form a smart city, integration with Edge computing is increasingly necessary to optimize bandwidth usage and reduce risks during data transmission [13].

The cloud offers nearly unlimited virtual resources and a wide spectrum of services [14]; however, applications that require fast, real-time processing may suffer, which is where the Edge computing paradigm comes into play. Despite the fact that the integration of Edge computing with Artificial Intelligence techniques can bring clear benefits [15] in optimizing and improving existing systems and workflows, Artificial Intelligence brings an extra layer of complexity to Edge computing [16] by minimizing data overload and delays in communication [17]. There are also certain aspects that can limit us, such as choosing an AI algorithm that can work optimally with the limited resources offered by Edge nodes, to enhance the efficiency [18]. The parallel use of artificial intelligence techniques together with IoT devices led in [19] to the definition of AIoT systems—AI-of-Things, systems that can provide a respond to an external input without human intervention. However, there is a need for human monitoring because processes can occur that can reach certain states and cannot be interpreted correctly. According to [20], comparing the results obtained from the integration of smart building/smart city with artificial intelligence techniques is quite difficult to achieve since there is no specific standard that applies to data sources, so that the results are comparable. Within smart buildings, artificial intelligence techniques are used for time series prediction, playing a particularly important role, especially for optimizing operating costs [21]. For resource utilization prediction, algorithms such as the one proposed by Sohani, M et al. [22] can be used to dynamically optimize this process with minimal impact on Quality of Service (QoS). Within smart cities, the variety and volume of devices and tests are very large, making resource allocation a rather difficult task to implement and optimize without an adaptive window optimization strategy [23] or similar. However, for a correct evaluation, several standard metrics such as CPU and memory usage can be used, gradually increasing the number of sensors and task length to observe and evaluate as correctly as possible the performance of the chosen techniques in various usage conditions; statistical approaches such as Mean Absolute Error (MAE) and Mean Absolute Percentage Error (MAPE) can also be used [24]. Depending on the chosen method, the performance may remain constant regardless of the scaling of the number of sensors [25].

This paper will present the advantages of using a solution that integrates Edge Computing technologies within smart buildings and smart cities, addressing an especially important problem within complex systems with dependencies between system nodes, namely, the dynamic allocation of resources to obtain optimal performance without manual intervention on the system. The data collected for this analysis is generated by sensors that monitor parameters such as air quality, temperature, and humidity inside the building; the focus of the paper is not the data itself but the way in which resources are allocated. Unlike existing techniques, the algorithm to be presented has as its main feature continuous training in order to provide information as relevant as possible for the next period of operation, so that resource allocation is made optimally, and tasks are divided as efficiently as possible between Edge and Cloud nodes. Although resource-consuming, continuous training ensures that the performance obtained will be optimal regardless of the usage scenario, operating hours, or the presence of an analyst to supervise the system. The more complex the prediction method chosen for better accuracy in air quality parameters, the more resource-consuming it is; for this reason, the model chosen for dynamic resource allocation is one that does not use resources intensively, even if it is trained continuously. Since the continuous training of production algorithms using artificial intelligence is resource-intensive, implementing such algorithms on a large scale within smart cities can prove to be a real challenge. The paper aims to bring performance improvements by dynamically allocating resources and moving process execution between Edge and Cloud based on CPU and RAM usage, the transition being performed automatically using the algorithm that will be presented, an algorithm that performs load prediction and performs calculations between Edge and Cloud.

The key findings and contributions of this paper can be summarized as follows:

- Continuously trained dynamic resource allocation framework: The proposed technique continuously learns from a 24 h window by analyzing the evolution of CPU and RAM usage. Based on the resource allocation algorithm, the system dynamically switches between cloud and edge nodes at key moments, adapting to changing workloads while maintaining stability.
- Predictive modeling and training: The SARIMA algorithm was employed to predict both CPU and RAM usage, and it consistently outperforms the baseline approach across s all four evaluated metrics: RMSE, MAE, MAPE, and MSE. To evaluate its performance under different conditions, testing was conducted in two scenarios: regular instance load and periods when a predictive Random Forest algorithm for air-quality parameters was triggered, introducing additional computational load. These tests demonstrate that the SARIMA model can maintain accurate CPU and RAM forecasts even under increased system demand, with minimal impact on ongoing processes.
- Robustness to critical scenarios: The approach addresses situations where cloud scalability is limited, demand is excessively high, or cloud resources are temporarily unavailable due to network failures. This ensures consistent and dependable operation in dynamic environments characterized by diverse devices and varying workloads.

The paper is organized as follows: In Section 2, a literature review will be presented, which will compare existing implementations and analyze the limitations and advantages of each implementation. Section 3 describes the proposed architecture, the implementation details of the proposed solution, thus presenting all the components used and the communication mode between them, followed by Section 4, to analyzes the experimental results obtained from the implementation of the solution and the comparison of the performance of this solution versus the use of a single Cloud/Edge component. The last section, Section 5, will bring us the conclusions of this implementation and the future steps.

The target audience of this paper comprises researchers, specialists, and advanced students with exposure to artificial intelligence techniques in the context of edge computing and dynamic resource allocation.

## 2. Related Work

In the context of the increasing volume of data generated by IoT devices, it is necessary to develop a robust solution that reduces the volume of data lost in critical moments, such as loss of internet connection, and allows the command loop to be closed for existing tasks even in these cases of failure, so that potential decisions are not impacted.

In this section, an analysis of existing implementations will be conducted, the methods used to improve performance inside smart buildings and cities, as well as analyzing the difficulties and problems encountered during implementations based on Edge computing and Machine Learning to streamline dynamic resource allocation.

Whether the discussion concerns smart buildings [26] or smart cities [27], starting from IoT devices to the servers that centralize them, modifying existing infrastructures through integration with edge computing is becoming an increasingly discussed topic, with a common goal regardless of the size of the system that is intended to be implemented, this goal being to improve performance by improving response time. To understand the direction of smart city development, several review articles were analyzed [28,29], where the problem of real-time data analysis is solved using Edge Computing, which solves some of the limitations of cloud resources, such as latency and network congestion.

In Walani, C.C. et al. [30] provide us with an overview of Cloud and Edge computing, in order to choose the desired technology depending on the specifics of the implementation, cloud for heavy load but with higher costs, or Edge for applications that need real-time processing with low latency. However, in smart cities, the complexity and variety of usage scenarios require a combination of the two paradigms, resulting in edge-cloud collaborative computing [31,32]; thus, the resource allocation has high complexity. Before choosing a specific algorithm for dynamic resource allocation, the results that are targeted by implementing such an approach must be defined; at this point, discussion can focus on reducing the costs necessary for the operation of the infrastructure [33], maintaining and improving Quality of Service while maintaining budget quotas [22], introducing task prioritization according to their importance [8], up to real-time monitoring and scheduling for the systems involved [34]. Depending on the scale of the implementation, the participating entities may be users who wish to offload tasks to the edge to minimize the resources they consume, and operators who need to manage requests and users through a joint communication and computation resource allocation mechanism [35].

In dynamic resource allocation, the most commonly used metrics that can be tracked to ensure the best possible QoS are, but are not limited to, CPU, RAM, storage, or bandwidth [36], but can also include the network and disk I/O throughput or the number of cores provisioned [24]. In the presented article, the focus is only on two parameters: CPU utilization and RAM memory utilization, because, since it is a real-time analysis, adding many metrics adds extra layers of complexity, thus increasing the time required to run the prediction, thus decreasing system performance.

One of the methods that can be used for dynamic resource allocation in smart cities is based on a 3-level architecture (IoT, Edge, and Cloud), the auction-based method, aiming to obtain optimal performance and reduced energy consumption [37]. An implementation using this method is proposed by Mahmood, O.A. et al. [38], which creates the desired allocation without considering the utility of the entities involved, the performance being influenced by the number of virtual machines involved in the system and the number of tasks, reaching an improvement of 9.2% compared to other methods.

Although the performances brought by Edge computing are indisputable, one particularly important aspect should not be overlooked: the way in which data is interpreted and the actions that can be taken based on the collected and processed data. Decision-making, especially real-time decision-making, automatically leads the discussion towards the introduction of artificial intelligence algorithms [39,40]. Very large AI models tend to be resource-intensive and often suffer from long response times. These issues can be addressed through pruning techniques, which eliminate unnecessary weights while maintaining prediction accuracy [41]. Thus, for smart cities, artificial intelligence techniques can be used to predict data and trends in mobility, security, water consumption, waste management or energy consumption [42], while also having an important role in the dynamic allocation of resources within cities, an implementation which according to [43] differs depending on the size of the cities, with better results for medium-sized cities (between one hundred and five hundred thousand inhabitants).

As in the works [44,45] in Table 1, the proposed work uses data generated by sensors regarding air parameters, but this can be extrapolated to several areas of use, such as waste management [46], energy prediction/management [47,48], or public safety [9]. Depending on the complexity of the chosen algorithm and the static training method, on a data sample or dynamic with continuous learning based on information transmitted by sensors, the resource utilization loading can be problematic, requiring optimization techniques [49] to obtain optimal results.

There is a very wide variety of parameters that can be considered to improve the efficiency of the system, regardless of whether the discussion concerns Edge, Cloud, or local resources. Table 2 shows the parameters used in existing works for the improvement of the efficiency of the system. There are cases such as [50,51] in which the evolution of a single resource, namely CPU, was followed, cases in which the performance analysis is desired depending on the CPU and memory load [24,52] but also very complex cases in which more specific parameters are introduced such as disk and network bandwidth [53], number of mobile nodes [54], SLA default rate and cost [31], load balance [55], or Local and Cloud Latency [8]. At the same time, the architecture used in terms of CPU and memory resources is also described, these being the parameters of interest for the current paper. Although the parameters of interest are countless, for the current paper, the decision was made to use only the most common metrics, present in most existing articles, namely CPU and RAM. The reason for this decision was the desire to avoid adding an extra layer of complexity to the dynamic allocation algorithm, complexity that automatically translates into higher resource consumption.

In order to compare the results obtained by the dynamic resource allocation algorithm and to have comparable results with other works, the metrics that can be used for the comparison must be defined. For this reason, in Table 3, the most used comparison metrics of the prediction errors of the proposed algorithms have been introduced, there are works such as [45] that use three of the four metrics considered, papers such as [11,23] that propose only two metrics or works such as [43,54] that use a single metric to analyze the results of the algorithm. Thus, it can be observed that some algorithms manage to obtain impressive results, with very small errors.

Analyzing the aggregated information from Table 1, Table 2, Table 3 and Table 4, it can be observed how the vast majority of existing articles address only two of the three objects of interest simultaneously, this being the main differentiator of the presented work, this work aiming at the prediction of air quality parameters, the dynamic allocation of resources between cloud and Edge as well as the evolution of the computer load.

## 3. Materials and Methods

This section will introduce the system components, the sensor part, the infrastructure used for the cloud and edge node, the benefits of using the Edge node to collect data transmitted by sensors, the presentation of the algorithm used to predict data collected from sensors, as well as the analysis of the dynamic resource allocation algorithm and the transfer of tasks between the Cloud and Edge nodes to obtain the best possible performance and the fastest possible response time for task execution.

The paper proposes the use of robust and scalable technologies designed to reduce data loss from IoT devices of interest. Increasing the volume of collected values leads to a better prediction of future values, with fewer gaps within the data sets. It is necessary to use a platform that has both Cloud and Edge capabilities, synchronized in real time. These dependencies are subsequently utilized to predict the system load, enabling dynamic resource allocation. In exceptional cases, such as increased latency, inability to provision the requested resources due to high demand, or elevated costs with cloud resources, the system ensures the transition of the command processing to the Edge node rather than the interruption of the execution process.

### 3.1. University Campus Infrastructure

**Figure 1:**
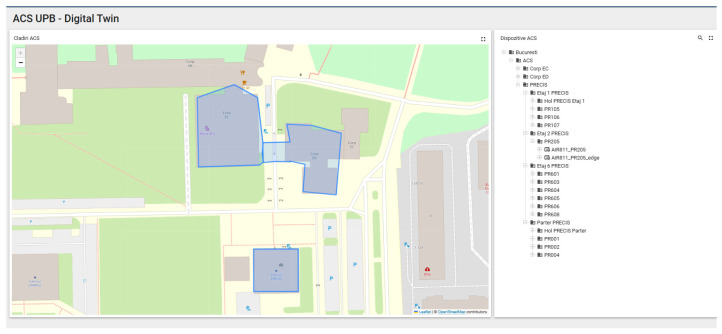
Presentation of buildings of interest within the campus and mapping of sensors.

The study has as its starting point the experimental platform digitaltwin.upb.ro. This platform encompasses three buildings on the university campus, with a total of 40 active sensors that generate data on the evolution of building parameters in real time. At the heart of this solution is the Thingsboard platform(ThingsBoard, Inc., New York, NY, USA), an open source platform dedicated to integrations with IoT devices, which in addition to integrating the ability to visualize data collected by various sensors installed in the faculty buildings, also offers us capabilities such as setting alarms, better infrastructure management, ease of integrating a multitude of IoT devices, as well as the existence of a REST API that adds versatility and scalability to the system. The Thingsboard platform offers us a robust, fault-tolerant architecture, adding customization capabilities to users and administrators through rule engine nodes and widgets. For message queue management, Thingsboard offers us two alternatives, these being Kafka and In-Memory, which can be used depending on the complexity of the system that is to be implemented. Thingsboard provides users with a wide range of specific libraries for microcontrollers, single-board computers, or other devices such as Gateways or LoraWan Devices [58]. Also, being an open source platform, the community’s contribution is felt through the constant addition of new functionalities, rapid integration with new devices, and rapid identification and resolution of any bugs that may appear for certain scenarios within the implementations. Figure 1 presents the three previously mentioned campus buildings as well as the detailed mapping of the 40 sensors. They are mapped according to the building block in which they are located, the floor, but also the room in which they are installed, each room having a unique number that helps us identify it more easily.

### 3.2. Quantitative Data Performance Comparison Between Edge and Cloud Nodes

On the current infrastructure, certain limitations were identified regarding the quality and quantity of the data collected, since during a reference time interval, there may be significant data losses, caused by external causes such as malfunctions of the campus internet network or power outages. These events can occur several times in 24 h and may have different recovery times depending on the cause of the incident. Thus, quantitative losses of data collected by sensors in the university campus buildings were identified.

Starting from this issue, the implementation of an Edge architecture (which is currently in the experimental phase) within the university campus is being evaluated to improve the quantitative collection of data and air monitoring infrastructure. Thus, the introduction of a new component within the existing system was experimentally achieved: the Edge node. Adding the Edge layer does not cover power outages, but it manages to fully cover network outages and packet loss during data transfer. Even if the internet connection is not available for a certain period of time, during malfunctions, until the situation is remedied, the Edge node operates, establishing the connection with the sensors via the local network inside the laboratory, saving the data generated by the sensors locally, and when the internet connection is restored, synchronization with the cloud node is performed. Immediately after synchronization, the data will be available both by accessing the edge node via the local network and directly on the cloud node, accessible regardless of the user’s location.

**Figure 2:**
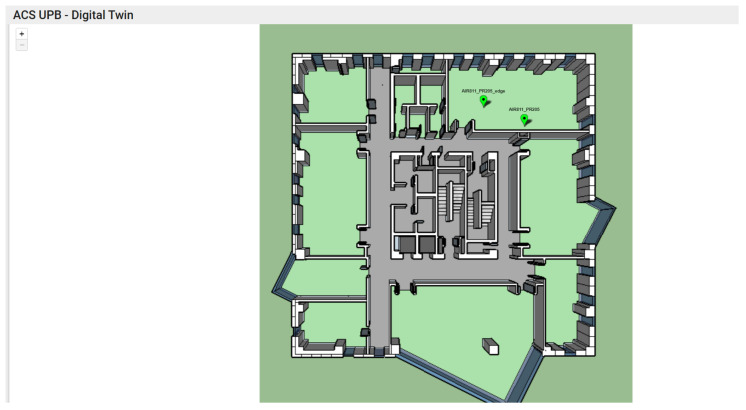
Allocation of the two sensors of interest inside the building and on the floor.

To demonstrate the improvement of the data collection capacity generated by the sensors by introducing an Edge node, we will compare the results of two identical sensors that generate air quality data, these being introduced within the PR205 laboratory in the PRECIS building of the university campus. The major difference between the two sensors is the way they are connected to the server that collects and processes the data. The first air quality monitoring sensor, called AIR811_PR205, is directly connected to the Cloud server, transmitting the data generated every 5 min via the internet connection. The second air quality monitoring sensor, called AIR811_PR205_edge, is highlighted by the direct connection to the Edge node, via the local network of the PR205 laboratory, the edge node being the one responsible for synchronizing the data with the Cloud node, via the internet connection. Figure 2 shows the location mapping where the two sensors were mounted inside the building, so we can better visualize the fact that the information transmitted by the two sensors, both in terms of quantity and in terms of the accuracy of transmitting information regarding air quality, should be similar.

### 3.3. Air Quality Parameter Prediction Algorithm

The two indoor air quality monitoring sensors in the PR205 laboratory, AIR811_PR205_edge and AIR811_PR205_edge, shown in Figure 2, generate the following metrics at 5 min intervals:

- Temperature;
- Humidity;
- VOC Index—Standardized index used to describe the level of volatile organic compounds in the air;
- PM2.5—Particulate Matter 2.5 measures airborne particles with a diameter of up to 2.5 μm;
- DeltaPm10—Variation in PM10 particle concentration between two successive measurements
- DeltaVOCI—Variation in VOC concentration in the reference period
- NOxi—Nitrogen Oxides Index indicates the concentration of Nitrogen Oxides in the air
- Pm1—Particulate Matter 1 measures airborne particles with a diameter of up to 1 μm;
- Pm2—Particulate Matter 2 measures airborne particles with a diameter of up to 2 μm;
- Pm4—Particulate Matter 4 measures airborne particles with a diameter of up to 4 μm;
- Pm10—Particulate Matter 10 measures airborne particles with a diameter of up to 10 μm.

To implement the air quality prediction algorithm, only four most commonly measured sensor metrics were used: temperature, humidity, VOC Index, and PM2.5, all of which displayed substantial variability during the experimental phase. As in [47], the chosen algorithm is Random Forest, a Machine Learning algorithm. Having a sufficiently large data set, the correct training of the algorithm can be performed, while it is also resource-consuming, making it perfect for implementing the dynamic allocation algorithm, loading the nodes enough to pose problems to a single instance.

The algorithm used for air parameter prediction, Random Forest, is based on several decision trees, each training on a different set of data; finally, a prediction is received from each tree, the final result being the average of the predictions of all trees. The operation of the algorithm can be divided into four individual steps:

1. Data preparation: the values generated by the sensors over a certain time interval are read, and based on them, lagged copies of the time series are built, also called lag features; these are stored in vector form, contain the values for the considered time interval. In other words, lag features are past values of the time series that are used to predict the future state.
2. Bootstrap Sampling: The data that can be used for each decision tree is randomly chosen. An important thing to note is that the data can be used by multiple trees.
3. Tree construction: Each tree is divided into several branches depending on the configured depth, with the possibility of stopping if it does not have enough data to share, thus reaching a final node, also called a leaf node.
4. Aggregation of results. Each tree sends a certain prediction; in this last step, all the predictions generated by the trees are aggregated, thus obtaining a more precise value compared to a single tree that presents a single way of placing the nodes.

By following these steps, some of the trees will find certain patterns and correlations between certain values, such as the evolution of temperature and humidity in the room. Since the algorithm is complex, it offers us great versatility in customization and configuration capabilities to improve the results obtained. The parameters used to configure and adjust the algorithm are described in Table 5.

**Figure 3:**
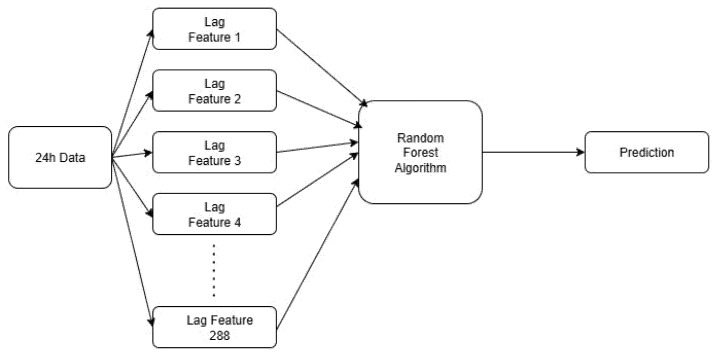
Random forest algorithm description.

The Random Forest algorithm used and detailed in Figure 3 is retrained for a period of 24 h, with data samples collected every 5 min, thus resulting in a set of 288 values, and manages to make the prediction for the considered interval of one hour. By using continuous retraining to achieve prediction, a number of 300 trees is used, a sufficiently large number for a more stable prediction, without the risk of overfitting by limiting the maximum depth of each component tree to only eight levels. The maximum depth of the trees is also influenced by the minimum number of samples, min_samples_split and min_samples_leaf. If there are not enough samples, the split is stopped at the current depth. For greater tree diversity and to reduce the correlation between trees, the max_features parameter was set to sqrt, so starting from the 288 values for the four air quality parameters considered, there is a total of 1152 features. Applying the sqrt function results in a max_features ~=34; in this way, out of the 1152 available features, only 34 will be considered at each split in a decision tree.

The complexity of the Random Forest algorithm is directly reflected in the resource usage, being a complex algorithm; the size of the data set influences the CPU and RAM load [58,59]. The most crucial factor that directly influences the degree of resource loading is the duration of training and prediction; a longer duration resulting in a higher resource usage.

Despite the fact that the main goal of the study is not the prediction of air quality parameters, but the dynamic allocation of resources between the Edge node and the Cloud node, the introduction of a time series prediction algorithm for parameters generated by air quality sensors is necessary in order to train and monitor the performance of the dynamic resource allocation algorithm on real data within a functional system.

### 3.4. Cloud Edge Switching

**Figure 4:**
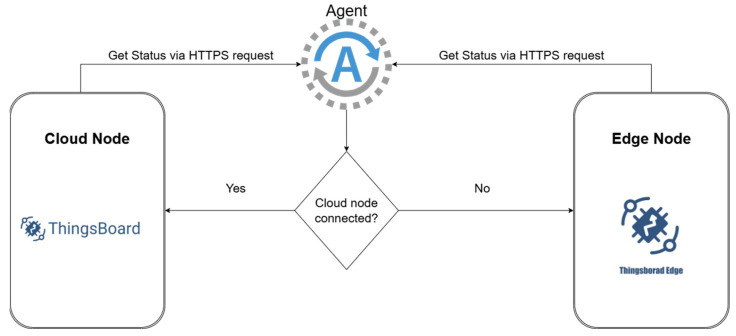
Resource switching diagram between Cloud and Edge.

In Section 3.2, a quantitative comparison of data collection between the Edge node and the Cloud node was presented, emphasizing the benefits of introducing an Edge node, a node that helps reduce data loss during internet connection interruptions. For an approach that uses only one Cloud node, the loss of internet connection or the connection between the sensor and the Cloud means not only a weaker collection of values during the incident, but even when a certain automation or alarm mechanism is running, it means the complete loss of information and the inability to complete the task that is currently being executed. By introducing the Edge node, this problem can be mitigated, with the switching between resources being the motivation underlying the implementation of the dynamic resource allocation algorithm. Figure 4 presents the switching logic between the two resources. A distinct agent is introduced that communicates with the Thingsboard platform. Through an HTTPS request, the agent checks the attributes of the two entities, assigning the connected or disconnected status to one of the nodes. This flag cannot have the same value for both nodes at the same time. Implicitly, if the cloud node is connected (1), the edge node will have the flag disconnected (0), and the parameter storing this value will be of type Boolean. This logic is described in Algorithm 1. In practice, this logic will transfer the execution of critical processes, such as alerts or existing automations, from the Cloud node to the Edge node until the internet connection is re-established. In this way, when the connection between the Edge and the Cloud is lost, the control loop is maintained through the Edge node, allowing the Random Forest prediction algorithm to continue operating locally. This ensures that the control mechanism can sustain the relevant system parameters within their defined reference ranges, even under network disruptions.
**Algorithm 1 Pseudocode: Resource switching between Cloud and Edge****Input:**         Status of the Cloud Node and Status of the Edge Node **Output:**         Decision in node selectionBEGIN     cloud_node_status = GET_STATUS (“Cloud Node status via HTTPS”)     edge_node_status = GET_STATUS (“Edge Node status via HTTPS”)     IF cloud_node_status == “CONNECTED” THEN         use_node(“Cloud Node”)     ELSE         use_node(“Edge Node”)     END IF END

The switching mechanism presented performs the switching only in case of failures, such as a lack of internet connection, having a further role of protecting the amount of data during the failure, the edge node playing only a backup role. Having as a starting point this switching between resources in case of failure, the aim was to improve the mechanism and introduce a new parameter to be considered for switching, which is the load of the nodes. By adding the load parameter, it is desired not only to cover failure cases but also to improve performance and the optimal execution of processes depending on the load of the considered resources. It is aimed not only at switching resources between the two nodes in case of failure but also at the dynamic allocation of resources between the nodes, and a prediction algorithm will be introduced and presented that will switch between the two nodes based on the data with which it is continuously trained, the change being made without external input, before the resources reach a very high level of utilization that would act as a bottleneck for the tasks that are being executed. In this way, failure cases are simultaneously covered, but also the system’s performance; these functionalities improve the system both for the failover area and for the performance area.

To ensure technical rigor and predictable performance, we model the offloading decision as a function of real-time network states. Unlike static configurations, our system employs an active probing mechanism that periodically evaluates the connection quality.

Let S(t) represent the system state at time t. The network conditions are monitored via a dedicated script that captures a vector of metrics, denoted as *M_net* (Equation (1)):

(1) $$M_{net}=\left(\alpha ,\lambda_{avg},\beta \right)$$

where

- α (alpha) represents Service Availability, a binary value {0, 1} determined by a successful TCP handshake on the IoT service port (1883).
- λavg (lambda) is the Average Latency (ms), derived from the Round-Trip Time (RTT) of continuous ICMP packets over a sliding window.
- β (beta) is the Throughput (Mbps), measured via active transmission tests over a fixed duration (using iPerf3).

The decision function D(Mnet) enables the offloading mechanism only when validity constraints are met. This logic corresponds to the conditional checks implemented in our validation script (Equation (2)):

(2) $$D\left(M_{net}\right)=\left\{\begin{array}{c} \mathrm{ExecuteCloud},if\alpha =1\;AND\;\lambda_{avg}<\lambda_{thresh}\;AND\;\beta >\beta_{thresh}\\ \mathrm{Execute Edge}\;,\;otherwise \end{array}\right.$$

**Figure 5:**
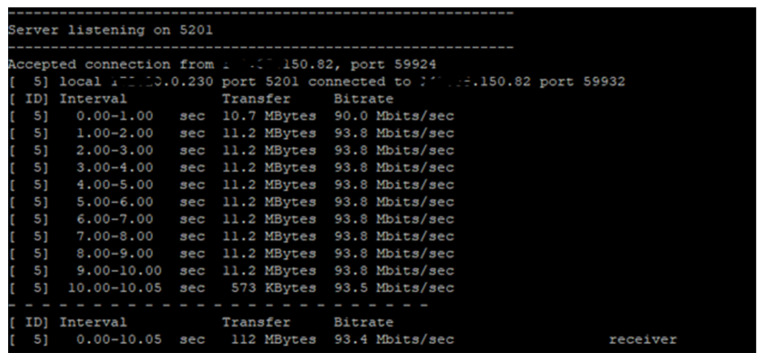
Testing the network between edge and cloud systems using iPerf3.

This mathematical model ensures that the system strictly adheres to the “Fail-Fast” principle: if the service port is unreachable (*α* = 0) or if latency exceeds the critical threshold (*λthresh*), the system defaults to local execution immediately, avoiding unnecessary timeouts. After testing the normal operation of the network between the edge machine and the cloud machine, *λthresh* = 1.8 ms and *βtresh* = 90 Mbit/sec (Figure 5) were established.

### 3.5. Infrastructure and Components Used

The infrastructure used to implement the dynamic resource allocation solution is based on the following components: the Cloud Node, the Edge Node, and the two sensors AIR811_PR205_edge and AIR811_PR205. Although the two sensors are similar, the major difference is the connection and communication method; AIR811_PR205_edge communicates directly with the edge node, without having direct communication with the cloud node, while for AIR811_PR205, the approach is different; it does not communicate with the Edge node, being directly connected to the Cloud node.

Table 6 details the Cloud node, which has a 4-core processor and 4 GB RAM. It runs the ThingsBoard open-source platform, using version 3.8.1, with the data volume stored in a PostgreSQL database version 12.22.

Table 7 details the Edge node based on a Raspberry Pi2B development board with a processor running at a maximum frequency of 900 Mhz, which, together with 1 GB of RAM, serves for the proper functioning of the ThingsBoard Edge platform in version 3.8.0, the data volume being stored in a PostgreSQL database in version 15.9.

Depending on the resources through which communication is carried out, the use and configuration of communication protocols differ. Table 8 including all these details; thus, the HTTPS protocols were used between the user and the Thingsboard platform, MQTT between the sensor and the Edge node, and RPC between the Edge node and the Cloud.

**Figure 6:**
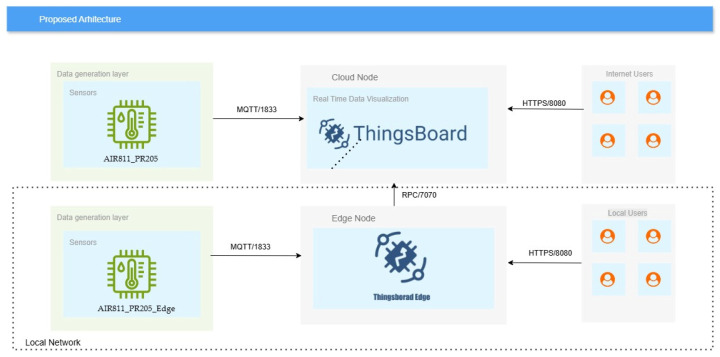
Diagram of the proposed infrastructure.

Figure 6 shows the architecture and the connection method between the components described above. Depending on the way in which users can connect to the Thingsboard/Thingsboard Edge platform, the proposed architecture can be divided into two categories: locally accessible and publicly accessible. The connection to the cloud node and implicitly the sensors attached to it can be made remotely by users regardless of their location via the HTTPS protocol and port 8080, being conditioned only by the existence of an internet connection. On the other hand, if there is no optimal internet connection, the components connected to the cloud node cannot be accessible, the connection being interrupted. In contrast, the Edge node is available, not depending on the existence of an internet connection, operating based on the local network, maintaining communication with the sensors directly connected to it, but it limits users by imposing location, requiring users to be present in the proximity of the Edge node in order to use the local network.

### 3.6. Dynamic Resource Allocation Algorithm

The main reason for switching between Cloud and Edge nodes is the loss of communication with the cloud node, regardless of the cause of this problem. Considering that based on the values collected from the sensors, certain control decisions are made that ensure the safety level of the building users, for example, the forced opening of the ventilation in case of detection of large quantities of Nitrogen Oxides, by switching the resources the values are not lost and the decisions can continue to be made, thus managing to close the control loop.

The main purpose of the dynamic resource allocation algorithm is to address exceptional cases, such as delays in the operation of cloud resource elasticity, resource scalability may be cumbersome, or, for financial reasons, accelerated load balancing brings increased costs. In this way, the introduction of the dynamic resource allocation algorithm can address such problems by performing resource prediction and switching to the Edge node, in order not to impact prediction, which can be the basis of a command. The algorithm aims to be able to predict the appropriate moment to move resources based on historical data, without waiting for the resource load to exceed a certain threshold to make this transition from one node to another. Thus, we can improve system performance by reducing processing times.

The prediction required for dynamic resource allocation must be performed by an algorithm that has the best possible performance with the lowest computational cost, thus limiting the impact on ongoing tasks. According to [60], the use of SARIMA brings very good results in prediction for short time intervals, being an optimal candidate for the dynamic resource allocation algorithm due to the low consumption of computational resources [61] required to provide the prediction, thus resulting in a minimal impact on other processes running concurrently. Thus, as in [24], SARIMA was chosen as the algorithm of interest, an algorithm that does not load resources very much and offers particularly superior performance for the desired time intervals, these performances being detailed in Section 4.

If the method illustrated in Figure 4 is employed to execute the query of the connection status of the nodes and performs the switch only in case there is no connection to the cloud node, by implementing the SARIMA algorithm, a new level of complexity is added that will cover not only possible connection problems but also the dynamic alternation between nodes depending on their usage. Considering that the interval for which we want to make the prediction is a short one, the algorithm offers increased efficiency with a minimal resource load.

The SARIMA algorithm is an extension of the ARIMA algorithm; the difference between the two is the addition of the seasonal component, which helps with the prediction of patterns that repeat regularly for the parameters of interest. In addition to the seasonal component, the algorithm has the following components: an AutoRegressive component, which determines the current value based on past values; an Integrated component, which is responsible for transforming the series by differences to make it stationary; and a Moving Average, which is responsible for determining the current value and based on errors in past predictions.

For the dynamic resource allocation between Cloud and Edge environments, focusing exclusively on CPU and RAM monitoring offers a pragmatic and efficient approach. These two parameters serve as primary indicators of computational capacity and memory, which are critical for predicting system performance. In contrast, adding additional parameters such as bandwidth, GPU utilization, or disk usage introduces significant complexity and variability without necessarily improving predictive accuracy. By limiting the monitoring to CPU and RAM, the allocation process becomes more robust and enables faster decision-making without a significant impact on the resources. Being continuously trained, the goal of the algorithm is to provide the best possible prediction without impacting existing processes by increasing resource utilization. Therefore, prioritizing CPU and RAM metrics provides a balanced trade-off between computational efficiency and predictive reliability.

The operation of the algorithm used for CPU and RAM prediction can be divided into eight individual steps:

1. Data collection and normalization—These are the CPU and RAM load data collected in the Thingsboard platform for the last 24 h, one hour before the current moment. In this step, the quality of the data is checked by checking the number of existing values and validating the 5 min frequency in data collection. The reason for choosing the 5 min interval for reading the data is to correlate with the moment of transmission of information regarding air quality, in order to better see the impact that this process has on resources. In case there are intervals with missing data, these values can be reindexed, or periods with very large gaps are eliminated.
2. Transformations—In this step, level variations are checked, and the stationarity of the series is verified.
3. Non-sensory and sensory differences—By applying the two components, the aim is to eliminate linear trends and the repetitive component.
4. Defining the model underlying the algorithm:

(3) $$\;(1-\Phi_{1}L^{12})(1-L)(1-L^{12})y_{t}=(1+\theta_{1}L)(1+{\Theta_{1}L}^{12})\varepsilon_{t}$$

where

$y_{t}$ represents the CPU or RAM value at the current time.

$L^{12}$ is the delay operator that takes the values one hour back (12 steps).

$\varepsilon_{t}$ represents the differences between the actual and predicted value, thus managing to capture unexpected fluctuations.

(1 − L) is the non-seasonal component that eliminates slow trends such as the constant increase in RAM load.

(1 + θL) is the non-seasonal component that adjusts the current prediction using the error of the previous step.

$(1-L^{12})$ is the seasonal component responsible for seasonal differences that eliminates hourly seasonality.

(1 − ΦL) is the seasonal component that uses the value from one hour ago to estimate the current value. If at the same time the CPU load value is high, this information is used.

$(1+{\Theta_{1}L}^{12})$ is the seasonal component that uses the prediction error to adjust the model.

5. Parameter estimation—Using numerical optimization to achieve the most stable fit.
6. Residuals—Checking the autocorrelation between the residuals, if it exists, the parameters must be adjusted.
7. Multi-step forecast—Using previous predictions to obtain more accurate values.
8. Post-processing—Additional steps may be applied for calibration or setting an offset.

Starting from Formula (3), the implemented algorithm groups the parameters into: non-seasonal parameters p, d, q responsible for short-term trends and dependencies, seasonal parameters P, D, Q, s responsible for repetitive patterns, as well as two additional parameters historic_days and prediction_hours intended to provide us with the necessary flexibility for setting the training time and the duration for which we want to make the prediction.

The selected SARIMA configuration (p, d, q) = (0, 1, 1) and {P, D, Q) = (1, 1, 1) with a length of seasonal cycle of 12 was based on an iterative and data-driven modeling process designed to identify a model that could accurately capture both the short term and repeatable structures present in the time series.

To ensure robustness, several alternative SARIMA configurations were evaluated around these initial estimates. Variations included adjusting the non-seasonal and seasonal autoregressive and moving-average orders, modifying the seasonal period to assess sensitivity to potential periodicity in the data. These alternatives included higher-order combinations and various levels of seasonal differencing to examine the model’s sensitivity to the underlying structure of the data. Each configuration was trained using identical historical sets of data and evaluated by the same metrics. Specifically, the following SARIMA configurations were explored:

- SARIMA (1, 1, 1) (1, 1, 1)
- SARIMA (0, 1, 1) (2, 1, 1)
- SARIMA (0, 1, 1) (1, 1, 0)
- SARIMA (0, 1, 2) (1, 1, 1)

However, all the alternatives produce higher RMSE, MAE, MAPE, MSE values compared to the final SARIMA model. Table 9 presents the values used to configure the SARIMA algorithm, values that were chosen after going through the eight steps.

**Figure 7:**
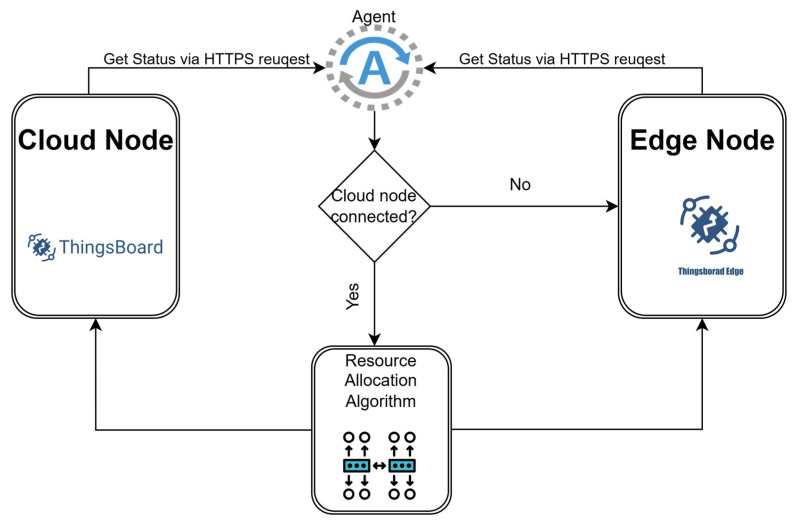
Resource switching diagram between Cloud and Edge based on Resource Utilization.

Unlike the implementation shown in Figure 4, Figure 7 introduces a new component, the predictive component. Thus, if both Cloud and Edge nodes are available, the primary node is no longer always the cloud node; the prediction algorithm that analyzes the resource load has the capability to transition tasks from one node to another based on the prediction for the next hour. Adding this new functionality does not affect in any way the scenario in which the Cloud node is not available, then the transition happens as before based on the output of the HTTPS request. This logic is described in Algorithm 2.
**Algorithm 2** Pseudocode: Resource switching between Cloud and Edge based on Resource Utilization**Input:**         Status of the Cloud Node and Status of the Edge Node         Resource Allocation Algorithm Decision **Output:**         Decision in node selectionBEGIN     cloud_node_status = GET_STATUS (“Cloud Node status via HTTPS”)     edge_node_status = GET_STATUS (“Edge Node status via HTTPS”)     IF cloud_node_status == “CONNECTED”         decision = ResourceAllocationAlgorithm (CPU, RAM)         IF decision == “USE_CLOUD” THEN             use_node (“Cloud Node”)         ELSE IF decision == “USE_EDGE” THEN             use_node (“Edge Node”)         ELSE             use_node (“Current Node”)         ENDIF     ELSE         use_node (“Edge Node”)     ENDIF END

## 4. Experimental Results

This section presents the quantitative results obtained following the introduction of the Edge node, including the performance of the random forest algorithm on air parameters, the impact of the algorithm on resource utilization, and the prediction performance of the SARIMA algorithm. Starting from the resource load prediction, the transition to the dynamic resource allocation algorithm will be re-evaluated.

### 4.1. Quantitative Data Performance Comparison Between Edge and Cloud Nodes

The quantitative performance comparison aims to highlight the improvements brought by the introduction of the Edge node in the number of values collected from the two sensors of interest. Better data collection can directly influence the quality of the prediction.

**Figure 8:**
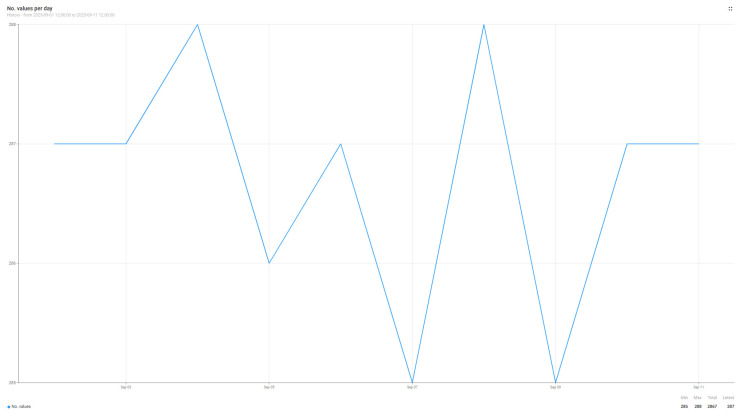
Number of values received from the AIR811_PR205_edge sensor by the Edge node.

**Figure 9:**
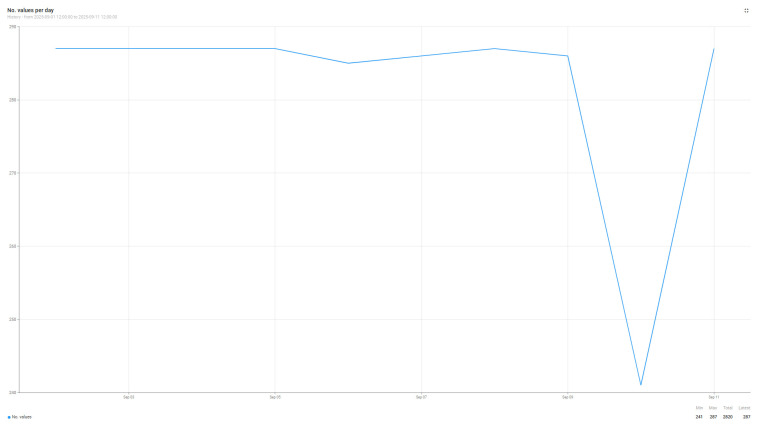
Number of values received from the AIR811_PR205 sensor by the Cloud node.

Figure 8 and Figure 9 show the number of air quality parameter readings transmitted by the sensors and received by the Edge Node (Figure 8) and the Cloud Node (Figure 9). By comparing the two graphs, we can analyze the differences between an implementation dependent on the internet connection, the cloud implementation, and the one in which we include the Edge node. In the case of the cloud implementation, there are certain times when, due to incidents at the network level within the campus, although the sensors generate data, they cannot be sent to the cloud node, and for certain time intervals, the data is completely lost. The data generation and transmission interval is 5 min for each of the sensors; every 5 min, an entry is generated to the cloud or edge node. The reference period for which the data were analyzed is 10 days between 1 and 11 September 2025, the period in which the AIR811_PR205 sensor generated 2820 entries, while the AIR811_PR205_edge sensor generated 2867 entries. The data in Figure 8 and Figure 9 have been summarized in Table 10; thus, in the reference period, the Edge node has a better performance by approximately 2% in data collection compared to the Cloud node. Although in percentage terms this improvement does not seem very large if we check specifically certain days in which network problems or functionality disturbances persisted for a longer period of time, an improvement of up to 16% in the number of data collected by the edge node vs. the cloud node is observed. On average, this improvement brought by the Edge node consists of adding five additional values to the existing series, thus reaching 287 daily values, versus the Cloud node, which collects only 282 daily values. For the considered time period, the Edge node loses only 13 values generated by the sensors, while the Cloud node loses 60 values; these losses ultimately boil down to errors in data interpretation.

### 4.2. Random Prediction Performance

**Figure 10:**
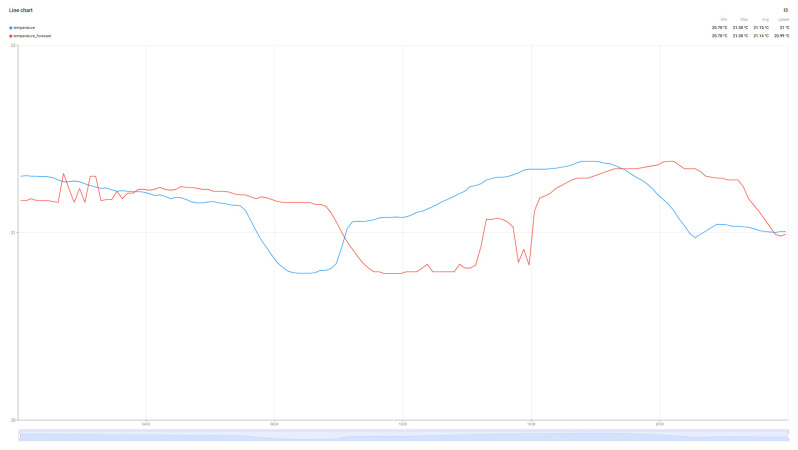
Temperature prediction forecast.

**Figure 11:**
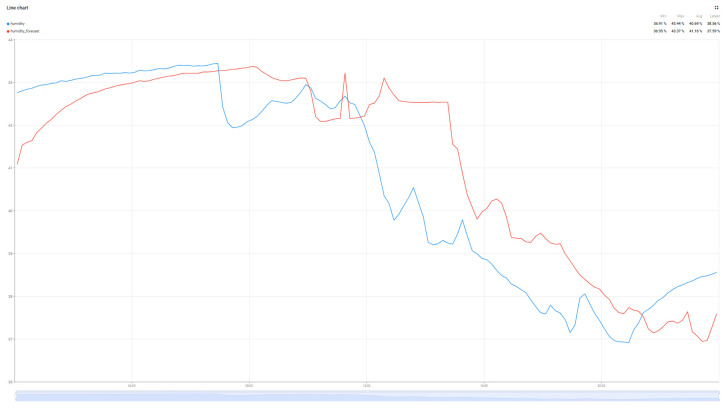
Humidity prediction forecast.

**Figure 12:**
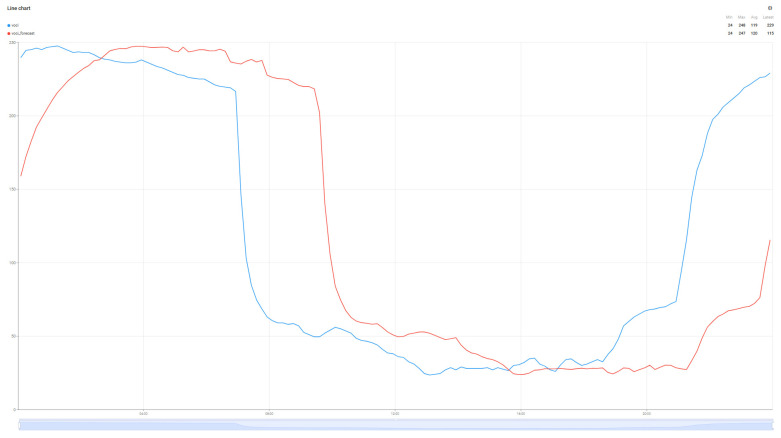
VOC index prediction forecast.

**Figure 13:**
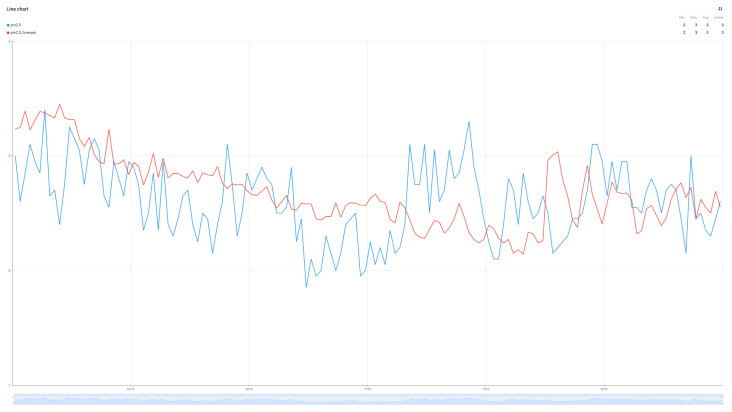
PM2.5 prediction forecast.

Although not the main purpose of the work, the evolution of air parameters for the data collected from the sensor was performed using the Random Forest Algorithm for the four collected metrics: Temperature, Humidity, VOC index, and PM2.5. The predictions for these values are detailed in Figure 10, Figure 11, Figure 12 and Figure 13.

The graphs in Figure 10, Figure 11, Figure 12 and Figure 13 present the prediction results over a 24 h period for the parameters of interest. Analyzing the average values transmitted by the sensors in the reference period with the prediction values of the Random Forest algorithm, minimal errors can be observed: Average real temperature: 21.15 °C, predicted temperature being 21.14 degrees Celsius; Average real humidity: 40.69%, predicted humidity being 41.16%; Average real VOC Index: 119, while the predicted VOC Index value is 120; The last parameter pm2.5 records the largest variations between the two values, the average of the real value being 4, while the average of the predicted value is 7.

### 4.3. Random Forest CPU and RAM Impact

**Figure 14:**
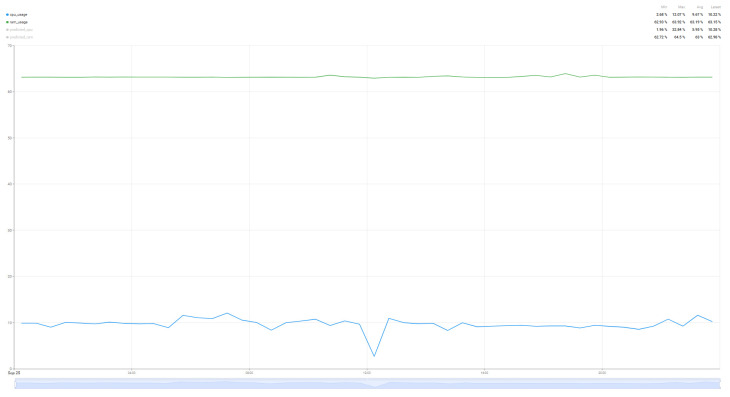
Resource usage (CPU and RAM) without the air quality prediction algorithm over a 24 h period.

**Figure 15:**
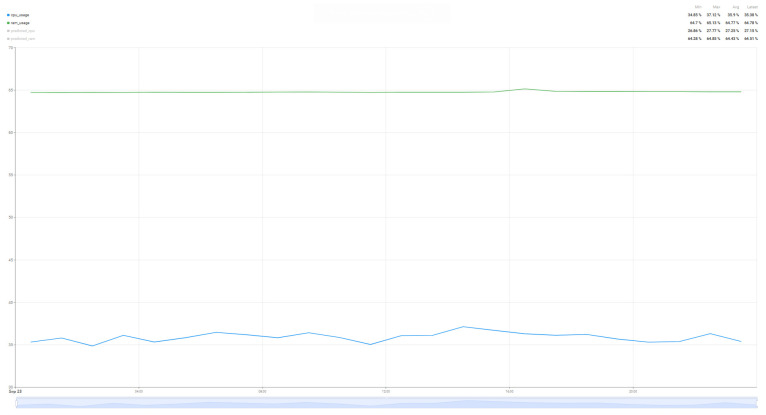
Resource usage (CPU and RAM) when the air quality prediction algorithm runs over a 24 h period.

In order to compare the degree of load that such an algorithm brings to the resources of interest (CPU and RAM), a comparison was made over a 24 h period of resource load for the case where the system operates without the prediction algorithm (Figure 14) and the system load when the air quality parameter prediction algorithm operates (Figure 15). In this way, a minimum baseline of resource usage could be set for the implemented system, without having as a direct objective the quality of the air quality parameter prediction, but rather how much the system requires such an algorithm.

Thus, analyzing the information presented in Figure 14 and Figure 15, we can see that the proposed algorithm brings an increase in CPU usage by 260%, from 10% utilization to up to 36%, while the increase in RAM memory usage is much smaller, of only 1.6% from 63% load to 64% load for a single sensor with four parameters that were taken into account in the chosen prediction algorithm. Thus, as demonstrated in [59,62] and in the case of the proposed implementation for air parameter prediction, the Random Forest algorithm increases CPU usage, but without a major impact on RAM memory due to the fact that the data set used and the prediction time are not very large.

By introducing the experimental prediction algorithm for air quality parameters, for only one sensor with four values of interest, the resource usage increases greatly, it can be easily assumed that for using the algorithm for the 40 existing sensors in the buildings on the university campus the load can be much higher and then sharing the effort with the experimental Edge level is a case that deserves to be explored.

### 4.4. SARIMA CPU and RAM Prediction

**Figure 16:**
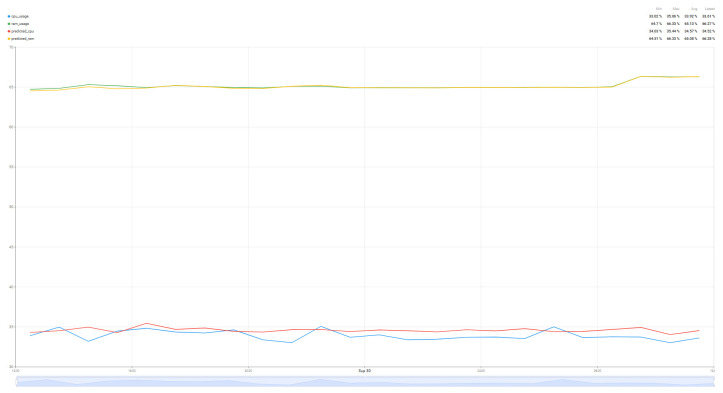
Resource usage (CPU and RAM) while the Random Forest algorithm is running.

**Figure 17:**
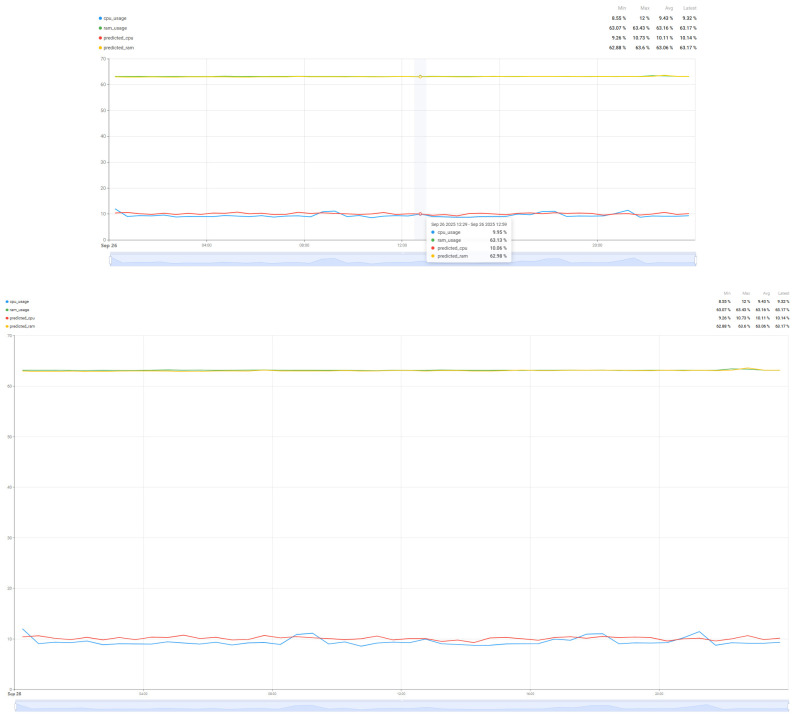
Resource usage (CPU and RAM) while the Random Forest algorithm is NOT running.

The introduction of the SARIMA algorithm aims to predict the evolution of the load of the resources of interest, CPU and RAM. Having a more accurate prediction of the values, the patterns of resource usage can be tracked, thus performing the migration of tasks between the two nodes. For a better comparison of the prediction results, two different scenarios were considered: Tracking the resource load prediction in the case where the air quality parameter prediction algorithm works (Figure 16) and in the case where the air quality parameter prediction algorithm is run on the Edge node or does not work (Figure 17). Thus, a difference in the quality of the prediction can be observed depending on the degree of resource load, especially for the CPU, with the RAM values being very stable, which gives us a much more accurate prediction. Although there are some differences in the performance of the two scenarios, the algorithm used is versatile enough to be used for multiple scenarios, being parameterized so that it can handle a wide range of resource usage and load scenarios.

Table 11 and Table 12 analyze the error evaluation metrics to measure how good the predictions offered by the SARIMA algorithm are for the parameters of interest, CPU and RAM, analyzing two scenarios: the period in which the air parameter prediction algorithm runs and the period in which the air prediction algorithm is switched to the Edge node, over a period of 24 h.

Analyzing the results for the case where the air prediction algorithm is running, the error is approximately 1.3–2 units or ~4% for the CPU, while the RAM prediction is 0.26–0.46 units, with an error of <1%. In the case where the air prediction algorithm is run by the Edge node or is not running, the prediction becomes even more accurate, the absolute deviations being significantly reduced from 2.13 to 1.02 for the CPU and from 0.45 to 0.15 for the RAM. Following the same trend, the average absolute errors are especially for the CPU from 1.33 to 0.95, but also for the RAM from 0.26 to 0.12. Another aspect that is improved is the variability of the error, which decreases a lot for both components. Although the absolute deviations have decreased, the relative error for the CPU increases from 4% to 10% while for the RAM it has decreased much more from 0.4% to 0.19%. Although at first the percentage value of 10% seems high, the explanation is quite simple. In this scenario, the CPU load is very low, about 10 units, so having an absolute error of 1.02 for the CPU, this translates into a 10% difference between the predicted value and the real value.

The baseline method used for comparison is the Simple Moving Average [63], a fundamental time series forecasting technique that estimates the next value by computing the arithmetic mean of a fixed number of past observations. This approach smooths short-term fluctuations and provides a simple reference model against which more advanced methods can be evaluated. The formula used to compute the Simple Moving Average for the comparative analysis is presented in Equation (4).

(4) yt+1=1i∑k=0i−1nkyt−k

where

- $y_{t+1}$ is the term to be predicted.
- $y_{t-k}$ is the observed value at lag k.
- i is the number of observations in the chosen time window.

The formula was applied to the two time intervals shown in Figure 16 and Figure 17, covering both usage scenarios: with and without the air parameters prediction algorithm. In this scenario, where the Random Forest algorithm runs, the implanted SARIMA algorithm improves CPU prediction accuracy by 26–45% and RAM prediction by 19–35% compared to the baseline method, depending on the metric used. In the scenario where the air parameters prediction algorithm does not run, CPU prediction improvements range from 19 to 64%, while RAM prediction remains stable, improving between 19–33%.

These consistent improvements demonstrate that the proposed SARIMA model significantly outperforms the baseline across all major error metrics, demonstrating substantially higher predictive accuracy for both CPU and RAM.

### 4.5. Dynamic Resource Allocation

It has been demonstrated that the prediction provided is constant regardless of the degree of resource utilization; the algorithm manages to capture this evolution, so it can be considered as a decision element within the dynamic resource allocation algorithm. Having the resource load prediction for the next hour, decisions can be made to switch between the cloud and edge nodes so that tasks can function optimally. For the moment, no specific threshold has been set for making the switch; the main focus is on achieving the prediction.

**Figure 18:**
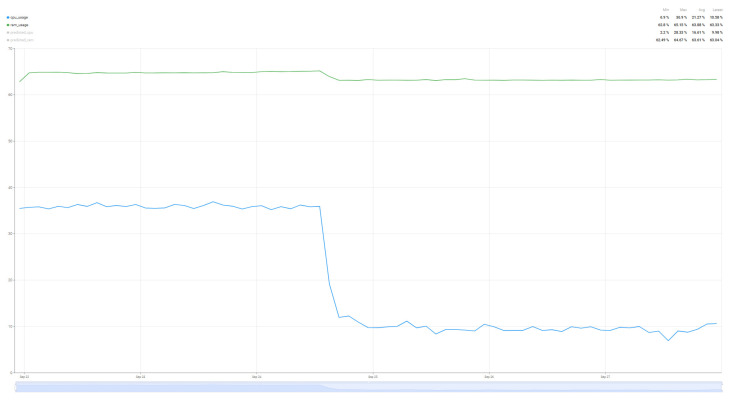
RAM and CPU utilization after EDGE switch.

Figure 18 shows the evolution of resource usage when the switch from the Cloud node to the Edge node was made dynamically. At the moment of losing the internet connection, the tasks being executed on the Edge node were paused until the connection was remedied.

By choosing a 24 h interval, the predictive algorithm captures a full resource usage cycle, which exhibits varying seasonal patterns depending on the time of the day. Maintaining an appropriate time interval allows enhanced performance while avoiding the resource burden associated with processing very long intervals.

Predictive performance for the next hour was selected to support resource management and real-time decision-making. Choosing a shorter interval would provide limited actionable insight, whereas longer intervals reduce forecast accuracy due to the inherent uncertainty in rapidly varying workloads.

In the case of a switchover caused by a lack of internet connection, checks are performed every 30 min to verify the availability of the cloud node. Once the cloud node is available and has sufficient resources, the switchover can be performed, with the Edge node load being transmitted to the cloud node.

**Figure 19:**
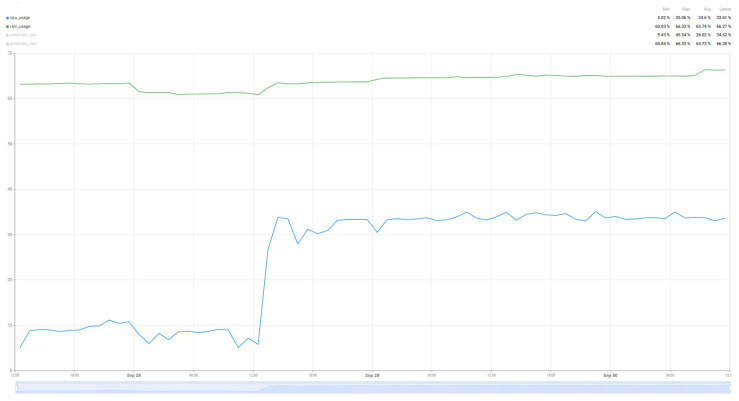
RAM and CPU utilization after connection is re-established and switch back to Cloud.

In case of switching caused by the load of the Cloud node, the tasks will be transmitted to the Edge node. To achieve optimal results, it is necessary to define the moment when the switching can be performed, and the rules defined for starting the re-evaluation of the load of the Cloud node. Figure 19 shows the moment when the switching between the Edge node and the Cloud node was performed, observing the increase in the resource usage load. In order to obtain the most conclusive results, but also to be able to prioritize the execution of tasks without performing too frequent switching between the two nodes, an interval of 30 min was used to perform the resource evaluation and make the decision to switch or continue using the current node. This value was selected to balance responsiveness with system stability. Shorter intervals could increase computational overhead and overreact to transient fluctuations, while longer intervals might cause delay adjustments to changing resource demands.

## 5. Conclusions

In this paper, the importance of using Edge computing for smart buildings/smart cities was presented, as well as the performance improvement by adding a dynamic resource allocation algorithm. The major contribution is represented by the implementation of switching between Edge and Cloud nodes, switching based on resource loading in the context of using artificial intelligence algorithms for predicting environmental parameters, the originality element being the dynamic resource allocation algorithm.

Taking as a starting point the Random Forest algorithm used for predicting air parameters and observing its impact on resource load, the improvement added by introducing an Edge node on the number of values collected from sensors was presented, managing to reduce the impact of external events such as loss of internet connection.

Adding the Edge node creates a robust system that can take the place of the cloud node in times when the internet connection is not available or in case the chosen cloud provider has problems in the availability zone where the resources have been deployed. Resource switching can be performed not only in case of such problems but also to optimize resource use and implicitly improve performance. In order to cover this dynamic resource allocation, the SARIMA algorithm was implemented, which, based on the CPU and RAM load history, predicts how the resource load will evolve. The results obtained from the implementation of the SARMA algorithm for predicting CPU and RAM load can be used as a decision element for achieving dynamic resource allocation.

While the experimental evaluation was conducted on a representative Edge-Cloud pair (one Raspberry Pi and one Cloud instance), this setup validates the atomic behavior of the proposed architecture. The monitoring and decision logic (implemented via the local control script) is fully decentralized. Each Edge node independently evaluates its network context (Mnet) and decides on task offloading without requiring peer-to-peer synchronization. Therefore, the functional validity of the offloading mechanism on a single node implies logical scalability to city-level deployments, assuming the cloud backend is provisioned to handle the aggregate load of concurrent connections.

Future research directions include examining the impact on key resources, such as CPU and RAM, to establish clear guidelines for determining an appropriate resource switching threshold based on the results of the resource usage prediction algorithm. This will require multiple tests over extended periods of time to optimize the transition from one node to another.

## Figures and Tables

**Table 1:** Related work: dynamic allocation of resources using Edge and Cloud computing for smart buildings and smart cities.

Author (s)	Algorithm	Scope	Smart Building	Smart City	Using Edge/Cloud	Using Dynamic Allocation of Resources
Luo, H. et al. [47]	Online Neural Network	Energy consumption prediction	✗	✓	✓	✗
Palagan, C.A. et al. [46]	RF algorithm and blockchain	Waste management	✗	✓	✓	✓ real-time data from IoT devices
Selvaraj, R. et al. [48]	AIMS-SB	Energy management	✓	✓	✗	✗
Wan, X. [49]	Federated Learning LO-DDPG algorithm	Cost reduction	✗	✓	✓	✓ optimization issues for the CPU frequencies, transmit power, IoT device offloading decisions
Zhao, H. et al. [9]	Back-propagation Neural Network (BPNN)	Public safety	✗	✓	✓	✗
Sun, C. et al. [44]	Deep-Learning	Air quality	✗	✗	✓	✗
Van Quang, T. et al. [45]	Artificial Neural Networks	Indoor air quality	✓	✗	✓	✗

**Table 2:** Related work overview of the used parameters for the resource allocation prediction and features.

Author (s)	Interest Parameters	Prediction	CPU	Mono-Processor Multi-Processor	RAM Memory	Virtual/Physical	Features
You, D. et al. [50]	Only CPU utilization	✓	✓	Not specified	✗	Not specified	Noise filtering
Anupama, K.C. et al. [24]	CPU and Memory utilization	✓	✓	Not specified	✓	Not specified	information of 1250 VMs- 30-day duration with the sample rate of 5 min
Tran, L. et al. [52]	CPU and Ram	✗	✓	16-core 2.1 GHz Intel(R) Xeon(R) Silver 4110	✓	48 GB DRAM	OS and applications coordinate to manage memory dynamically manages large volumes of sensor data
Shen, X. et al. [51]	CPU	✗	✓	2.4 GHz CPU	✗	16 GB	Analyzing the performance with different numbers of task between 10 and 150
Tuli, S. et al. [53]	CPU, RAM and disk, and network bandwidth	✓	✓	Intel i7-7700K	✓	8 GB graphics RAM	Looking at the number of tasks completed and the avg response time
Deng, X. et al. [55]	CPU Latency fairness, and load balance	✓	✓	Not specified	✗	Not specified	The number of tasks per server varies
Pettorali, M. et al. [54]	CPU Memory, number of mobile nodes	✓	✓	Different scenarios, 1 to 5 GHz	✓	8 GB	Considers the number of mobile nodes in the system
Lai, P. et al. [36]	CPU, RAM, storage, bandwidth	✗	✓	i5-7400T processor (4 CPUs, 2.4 GHz)	✓	8 GB RAM	quality of service and quality of experience
Anand, J. et al. [8]	CPU RAM Local and Cloud Latency	✓	✓	[500, 3000] Edge MIPS [2000, 10,000] Cloud MIPS	✓	Edge Node 32 Gb Cloud Node 64 GB	Time, execution time, and energy consumption
Li, C. et al. [31]	CPU, number of rented nodes, SLA default rate, and cost	✓	✓	Single Multi-Core comparison	✗	Up to 32, depending on the configuration	Comparing the performance/costs of 12 different configurations

**Table 3:** Related work: prediction algorithm and metric evaluation.

Author (s)	Prediction Algorithm	RMSE	MAE	MAPE	MSE
You, D. et al. [50]	CEEMDAN-LSTM-RIDGE	✓ 1.3141	0.8966 ✓	3.2030 ✓	✗
Palagan, C.A. et al. [46]	Random Forest	✗	0.08 ✓	✗	0.12 ✓
Kothamali, P.R. et al. [12]	Adaptive Resilient Node (ARN) Coordination Algorithm	Different values depending on the scenario ✓	Different values depending on the scenario ✓	✗	✗
Anupama, K.C. et al. [24]	SARIMA	✗	5.83 for CPU, higher values for memory prediction ✓	0.49 for CPU, higher values for memory prediction ✓	✗
Zhang, H. et al. [56]	Neural Network (CNN-BiLSTM)	✗	0.2387 ✓	✗	0.2222 ✓
Luo, H. et al. [47]	Online Neural Network	✗	✗	✗	Only graphical interpretation, no exact values ✓
Wu, H. et al. [57]	Distributed Deep Learning-Driven Task Offloading (DDTO)	✗	✗	✗	Not specified ✓

**Table 4:** Consolidated comparative summary.

Author (s)	Parameter Prediction for Smart City/Smart Building	Edge Computing with Dynamic Allocation	Prediction on the Evolution of the Computer Load
Luo, H. et al. [47]	✓	✗	✗
Palagan, C.A. et al. [46]	✓	✓	✗
You, D. et al. [50]	✗	✗	✓ error correction and data denoising
Zhang, H. et al. [56]	✓	✗	✓ real-time power prediction based on resource utilization
Anupama, K.C. et al. [24]	✗	✗	✓ Hibrid prediction, statistical + machine learning
Anand, J. et al. [8]	✗	✓	✓
Ali, A. et al. [12]	✓	✓	✗
Selvaraj, R. et al. [48]	✓	✗	✗
Zhang, Y. et al. [32]	✗	✓	✓ time-sensitive scheduling and priority algorithms
Ding, S. et al. [33]	✗	✓	✗
Pettorali, M. et al. [54]	✗	✓	✓ for real-time application
Romero, D.A. et al. [26]	✓	✗	✗
Our Solution	✓	✓	✓

**Table 5:** Random forest algorithm configuration.

Parameter	Description	Value
N_estimators	Number of trees used	300
max_depth	Maximum depth of trees	8
min_samples_split	Minimum number of samples in node for division	3
min_samples_leaf	Minimum number of samples from an end node/leaf node	2
max_features	Maximum number of features considered for each split	sqrt
n_lags	Lag copies of a time series	288
historic_days	Number of days used for prediction	1
prediction_hours	The number of hours for which the prediction is made	1

**Table 6:** Cloud node description.

Resource	Description
Processor	Intel(R) Xeon(R) CPU E5-2660 v2 @ 2.20 GHz, 4 cores
Memory	4 GB RAM
Operating System	CentOS Stream Linux 8
Kernel	kernel-4.18.0-553.6.1.el8.x86_64
ThingsBoard	3.8.1
Database	PostgreSQL version 12.22
Java OpenJDK	17.0.12

**Table 7:** Description of the components used for the Edge node.

Resource	Description
Development Board	Raspberry Pi 2B
Processor	ARM Cortex-A7 @ 900 MHz
Memory	1 GB RAM
Operating System	Raspbian Linux 12
Kernel	6.6.62 + rpt-rpi-v7
ThingsBoard Edge	3.8.0
Database	PostgreSQL 15.9
Java OpenJDK	17.0.13

**Table 8:** Description of communication protocols between resources.

Connection Between	Protocol	Port
Users and Platform	HTTPS	8080
Sensors and Edge	MQTT	1833
Edge node and Cloud	RPC	7070
Sensors and Cloud	MQTT	1833

**Table 9:** SARIMA algorithm configuration.

Parameter	Description	Value
p	Non-seasonal autoregression	0
d	Non-seasonal differentiation	1
q	Non-seasonal Moving Average	1
P	Seasonal autoregression	1
D	Seasonal differentiation	1
Q	Seasonal Moving Average	1
s	Length of the seasonal cycle	12
historic_days	Number of days used for training	1
prediction_hours	The number of hours for which the prediction is made	1

**Table 10:** Comparison of the number of values transmitted by sensors to Edge and Cloud nodes for a reference period of 10 days.

Metric	Cloud Node	Edge Node	Maximum
Min	241	285	288
Max	287	288	288
AVG	282	287	288
Total	2820	2867	2880

**Table 11:** SARIMA model results obtained while performing the air quality prediction over a period of 24 h.

Metric	CPU	CPU (Baseline)	RAM	RAM (Baseline)	CPU Improvement (%)	RAM Improvement (%)
RMSE	2.135454977	2.896112677	0.45163613	0.562766783	26.264	19.747
MAE	1.331007752	1.775537634	0.262015504	0.363548387	25.036	27.928
MAPE	4.285011928	5.546733386	0.409304104	0.565485126	22.747	27.618
MSE	4.560167959	8.387468638	0.203975194	0.316706452	45.631	35.594

**Table 12:** SARIMA model results generated without the air quality prediction over 24 h.

Metric	CPU	CPU (Baseline)	RAM	RAM (Baseline)	CPU Improvement (%)	RAM Improvement (%)
RMSE	1.025028997	1.718717676	0.149816348	0.185563462	40.360	19.264
MAE	0.945694444	1.493454936	0.122361111	0.164141631	36.677	25.453
MAPE	10.23814828	12.79209577	0.19367763	0.259492897	19.965	25.363
MSE	1.054600463	2.953990451	0.023613889	0.034433798	64.299	31.422

## Data Availability

The original contributions presented in this study are included in the article. Further inquiries can be directed to the corresponding author.

## Abbreviations

The following abbreviations are used in this manuscript:

RMSE	Root Mean Squared Error
MAE	Mean Average Error
MAPE	Mean Absolute Percentage Error
MSE	Mean Squared Error
VOC Index	Volatile Organic Compounds Index
LSTM	Long Short-Term Memory
SARIMA	Seasonal Auto Regressive Integrated Moving Average
SMA	Simple Moving Average
